# Identification of Chemotaxis Sensory Proteins for Amino Acids in *Pseudomonas fluorescens* Pf0-1 and Their Involvement in Chemotaxis to Tomato Root Exudate and Root Colonization

**DOI:** 10.1264/jsme2.ME12005

**Published:** 2012-09-05

**Authors:** Shota Oku, Ayaka Komatsu, Takahisa Tajima, Yutaka Nakashimada, Junichi Kato

**Affiliations:** 1 Department of Molecular Biotechnology, Graduate School of Advanced Sciences of Matter, Hiroshima University, 1–3–1 Kagamiyama, Higashi-Hiroshima, Hiroshima 739–8530, Japan

**Keywords:** plant growth-promoting rhizobacteria, chemotaxis, methyl-accepting chemotaxis protein, *Pseudomonas fluorescens*, root colonization

## Abstract

*Pseudomonas fluorescens* Pf0-1 showed positive chemotactic responses toward 20 commonly-occurring l-amino acids. Genomic analysis revealed that *P. fluorescens* Pf0-1 possesses three genes (Pfl01_0124, Pfl01_0354, and Pfl01_4431) homologous to the *Pseudomonas aeruginosa* PAO1 *pctA* gene, which has been identified as a chemotaxis sensory protein for amino acids. When Pf01_4431, Pfl01_0124, and Pfl01_0354 were introduced into the *pctA pctB pctC* triple mutant of *P. aeruginosa* PAO1, a mutant defective in chemotaxis to amino acids, its transformants showed chemotactic responses to 18, 16, and one amino acid, respectively. This result suggests that Pf01_4431, Pfl01_0124, and Pfl01_0354 are chemotaxis sensory proteins for amino acids and their genes were designated *ctaA*, *ctaB*, and *ctaC*, respectively. The *ctaA ctaB ctaC* triple mutant of *P. fluorescens* Pf0-1 showed only weak responses to Cys and Pro but no responses to the other 18 amino acids, indicating that CtaA, CtaB, and CtaC are major chemotaxis sensory proteins in *P. fluorescens* Pf0-1. Tomato root colonization by *P. fluorescens* strains was analyzed by gnotobiotic competitive root colonization assay. It was found that *ctaA ctaB ctaC* mutant was less competitive than the wild-type strain, suggesting that chemotaxis to amino acids, major components of root exudate, has an important role in root colonization by *P. fluorescens* Pf0-1. The *ctaA ctaB ctaC* triple mutant was more competitive than the *cheA* mutant of *P. fluorescens* Pf0-1, which is non-chemotactic, but motile. This result suggests that chemoattractants other than amino acids are also involved in root colonization by *P. fluorescens* Pf0-1.

Certain strains of *Pseudomonas fluorescens* exert beneficial effects on plants (3, 13, 17). These strains are able to enhance plant growth indirectly by preventing the growth or action of plant-pathogenic microorganisms. *P. fluorescens* F113 protects sugar beet seedlings from damping-off disease caused by the fungal pathogen *Pythium ultimum* by producing the antifungal metabolite 2,4-diacetylphloroglucinol, hydrogen cyanide, and extracellular protease (1, 22). *P. fluorescens* strain 2–79 is able to suppress wheat take-all produced by *Gaeumannomyces graminis* var. *tritici* by means of producing antibiotics phenazine-1-carboxylic acid (32, 33). *P. fluorescens* WCS365 is a biocontrol agent against *Fusarium oxysporum*, which causes tomato foot and root rot (4). This strain is an efficient root colonizer and it is assumed that it can favorably compete for available habitable niches on a root surface with the pathogenic fungus.

Efficient root colonization by plant-beneficial rhizobacteria is assumed to be essential for the biocontrol of root pathogens (30). In previous studies, it was demonstrated that motility and chemotaxis are important traits for root colonization by *P. fluorescens*. Simons *et al.* reported that a mutant of *P. fluorescens* WCS365, lacking flagella was outcompeted in the root-tip colonization assay when inoculated in competition with the parental WCS365 strain (24). Conversely, Barahona *et al.* showed that a hypermotile mutant of *P. fluorescens* F113, was more competitive for rhizosphere colonization than the wild-type strain and exhibited improved biocontrol activity against the pathogenic fungus *F. oxysporum* and the pathogenic oomycete *Phytophthora cactorum* compared with the wild type strain (3). de Weert *et al.* demonstrated that *cheA* mutant of *P. fluorescens* WCS365, which is non-chemotactic but motile, colonized the tomato root tip less efficiently than the wild-type strain in the competitive root colonization assay (6).

Plant root exudates contain various organic compounds. Major components of tomato root exudate are amino acids (glutamic acid, aspartic acid, leucine, isoleucine, and lysine as major components [25]), organic acids (especially citric acid, malic acid and succinic acid [10]), and sugars (glucose and xylose as major components [14]). Previous studies demonstrated that *P. fluorescens* strains exhibit chemotactic responses toward plant seed and root exudates and their components (6, 20, 26, 30); therefore, it is supposed that chemotaxis to components of plant root exudates is involved in effective root colonization.

Methyl-accepting chemotaxis proteins (MCPs) are chemotaxis sensory proteins responsible for the detection of chemotactic ligands (11). MCPs are membrane-spanning homodimers and typical features of MCPs are as follows: a positively charged N terminus followed by a hydrophobic membrane-spanning region, a hydrophilic periplasmic domain, a second hydrophobic membrane-spanning region and a hydrophilic cytoplasmic domain (7). Chemotactic ligands bind to periplasmic domains of MCPs and their binding initiates chemotaxis signaling. The diverse ligand specificities among MCPs reflect amino acid sequence diversities of periplasmic domains of MCPs. The C-terminal cytoplasmic domains of MCPs are relatively conserved. A 44-amino-acid highly conserved domain (HCD) is located in the cytoplasmic domain. MCPs from phylogenetically diverse bacteria have been shown to possess HCD (34). Blastp analysis found that in *P. fluorescens* Pf0-1 (accession number: CP000094), Pf- 5 (accession number: CP000076), and SBW25 (accession number: AM181176) genomes, there are 37, 34, and 46 gene products possessing HCD, respectively, and all have been annotated as MCPs; however, it is still unknown which exudate components and MCPs are involved in efficient root colonization by *P. fluorescens*. In the present study, we identified and characterized MCPs for amino acids, major components of plant root exudates, in *P. fluorescens* Pf0-1 and investigated their involvement in chemotaxis toward tomato root exudate and root colonization.

## Materials and Methods

### Bacterial strains, plasmids, and growth conditions

Bacterial strains and plasmids used in this study are listed in Table 1. *Escherichia coli* JM109 (18) and S17-1 (23) were used for plasmid construction and transconjugation, respectively. *P. fluorescens*, *Pseudomonas aeruginosa* and *E. coli* strains were grown with shaking in 2× YT medium (18) supplemented with appropriate antibiotics. *P. aeruginosa* and *E. coli* strains were cultivated at 37°C, while *P. fluorescens* strains were grown at 28°C.

### Chemotaxis assay

The computer-assisted capillary assay method was carried out as described previously (16). Cells in a 10 μL suspension were placed on a coverslip, and the assay was started by placing the coverslip upside down on the U-shaped spacer to fill the chemotaxis chamber with the cell suspension. Cells were videotaped over 3 min. Digital image processing was used to count the number of bacteria accumulating toward the mouth of a capillary containing a known concentration of an attractant plus 1% (w/v) agarose. The strength of the chemotactic response was determined by the number of bacterial cell per frame. The chemotaxis buffer was 10 mM HEPES (*N*-2-hydroxyethylpiperazine-*N*′-ethanesulfonic acid) buffer (pH 7.0).

### DNA manipulation

Standard procedures were used for plasmid DNA preparations, restriction enzyme digestions, ligations, transformations, and agarose gel electrophoresis (18). PCR reactions were carried out using KOD Plus DNA polymerase (Toyobo, Tokyo, Japan) according to the manufacturer’s instructions. Oligonucleotides used for PCR are listed in Table 2. *P. aeruginosa* was transformed by electroporation as described previously (15). Plasmids were introduced to *P. fluorescens* strains by transconjugation using *E. coli* S17-1 (23).

### Plasmid construction and construction of deletion mutants of *P. fluorescens* Pf0-1

The Pfl01_0124, Pfl01_0354, and Pfl01_4431 genes were amplified from *P. fluorescens* Pf0-1 genome by PCR using PFL01f/PFL01r, PFL02f/PFL02r, and PFL03f/PFL03r primer sets, and cloned into broad-host-range plasmid pUCP18 (21) to construct pFLCP01, pFLCP02, and pFLCP03, respectively. Suicide plasmids pUGMF01, pUGMF02, and pUGMF03 were constructed for unmarked gene deletion in *P. fluorescens* Pf0-1. PCR using primer sets DPFL01Uf/DPFL01Ur and DPFL01Df/DPFL01Dr was conducted to amplify 1.2-kb regions upstream and downstream of Pfl01_0124 from the *P. fluorescens* Pf0-1 genome, respectively. Amplified upstream and downstream regions were digested with *Pst*I-*Bam*HI and *Bam*HI-*Eco*RI, respectively, and ligated with the backbone of *Pst*I-*Eco*RI-digested pK18*mobsacB* (19) to obtain pUGMF01. PCR using primer sets DPFL02Uf/DPFL02Ur and DPFL02Df/DPFL02Dr was conducted to amplify a 1.4-kb upstream region and a 1.3-kb downstream region of Pfl01_0354 from the *P. fluorescens* Pf0-1 genome, respectively. Amplified upstream and downstream regions were digested with *Sal*I-*Bam*HI and *Sph*I-*Sal*I, respectively, and ligated with the backbone of *Sph*I-*Bam*HI-digested pK18*mobsacB* to obtain pUGMF02. PCR using primer sets DPFL03Uf/DPFL03Ur and DPFL03Df/DPFL03Dr was conducted to amplify a 1.3-kb upstream region and a 1.2-kb downstream region of Pfl01_0354 from the *P. fluorescens* Pf0-1 genome, respectively. Amplified upstream and downstream regions were digested with *Hin*dIII-*Xho*I and *Xho*I-*Eco*RI, respectively, and ligated with the backbone of *Hin*dIII-*Eco*RI-digested pK18*mobsacB* to obtain pUGMF03. The chromosomal Pfl01_0124, Pfl01_0354, and Pfl01_4431 genes were deleted by the unmarked gene deletion technique (19) using suicide plasmids pUGMF01, pUGMF02, and pUGMF03, respectively. Unmarked gene deletion was confirmed by PCR using PCR primers specific to upstream and downstream sites of each gene.

### Preparation of tomato root exudate

Exudate was prepared from a plant species, tomato (*Solanum lycopersicum* cv. Oogatahukuju). Tomato seeds were sterilized by gentle shaking for 10 min in a solution of 8.75% (v/v) sodium hypochloride supplemented with 0.1% (v/v) Tween 20. The sterilized seeds were soaked six times for 15 min in sterile demineralized water. Nine sterile seeds were placed in 3 mL SSE medium (2), consisting of 5 μM KH_2_PO_4_, 4 mM CaSO_4_, 2 mM MgCl_2_, 2.5 mM NH_4_NO_3_, 0.5 mM KOH, 2.5 mM NaOH, and 0.02 mM Fe (as FeEDTA), and were allowed to grow in a climate-controlled growth chamber (NK System, Osaka, Japan) at 28°C, and 16 h of daylight. After 18 days, root exudates were collected and evaporated to dryness at 45°C under a vacuum, dissolved in 1 mL water, and sterilized by membrane filtration (0.45-μm pore size).

### Selection for rifampicin resistance mutants

Spontaneous rifampicin-resistant mutants of *P. fluorescens* were generated by spreading bacterial cells, grown overnight in 2× YT, onto 2× YT agar plates containing 20 μg mL^−1^ rifampicin. The plates were incubated at 28°C for 20 h to form colonies. The resulting rifampicin-resistant colonies were then streaked on 2× YT agar containing 50 μg mL^−1^ rifampicin, and Rif^r^ strains were subsequently maintained on this medium. One mutant showing a growth rate similar to that of the wild-type strain was selected and designated Pf0-1Rif. Similarly, a rifampicin-resistant mutant of *P. fluorescens* FLD3 was obtained and designated FLD3Rif.

### Gnotobiotic root colonization assays

Forty grams of quartz sand (0.1 to 0.3 mm) was placed in glass tubes (22 mm inner diameter, 25 mm outer diameter, 12 cm length) and compacted by gentle shaking. The open end of the tube was plugged with a silicone resin stopper. The tube was then autoclaved for 15 min at 121°C. Ten milliliters of PNS (plant nutrient solution) (8), consisting of 1.25 mM Ca(NO_3_)_2_, 1.25 mM KNO_3_, 0.5 mM MgSO_4_, 0.25 mM KH_2_PO_4_, and trace elements (in mg L^−1^): Fe (as FeEDTA), 4.6; B, 0.5; Zn, 0.05; Cu, 0.02; Mo, 0.01, was added to an autoclaved sand column. Tomato seeds (*S. lycopersicum* cv. Oogatahukuju) were sterilized as described at “*Preparation of tomato root exudate*” section. To synchronize germination, seeds were placed on Petri dishes containing PNS solidified with 1.5% (w/v) Bacto Agar (Becton, Dickinson and Company, Franklin Lakes, NJ, USA) and incubated overnight in the dark at 4°C, followed by incubation at 28°C for 2 days. A germinated seed was aseptically placed at the center of a growth tube and 5 mm below the surface of quartz sand. Bacterial cells were grown for 14 h in 2× YT medium, centrifuged (3,300×*g*, 2 min), washed three times with PNS, and adjusted to 10^7^ CFU mL^−1^ in PNS. For the colonization assay, 100 μL bacterial cell suspensions were added to the edge of a plant growth tube. For the competitive colonization assay, 100 μL of 1:1 (v/v) mixture of the tested strain and the competitor were mixed and inoculated at the edge of a plant growth tube. The plant growth tubes were incubated in a climate-controlled growth chamber (28°C, 16 h daylight) to allow the plantlets to grow. After 7 days of growth in the plant growth tubes, the root systems of tomato were mostly unbranched. The root tip (1 to 2 cm length) was removed and shaken vigorously in the presence of adhering sand particles in 0.5 mL of PNS to remove bacteria. The bacterial suspension was diluted and 100 μL was plated on 2× YT agar plates. For the competitive colonization assay, the bacterial suspension was spread on 2× YT agar plates with and without rifampicin. For statistical analysis, the nonparametric Wilcoxon-Mann-Whitney test was used (27).

## Results

### Chemotactic responses toward amino acids by *P. fluorescens* Pf0-1

Amino acids are one of the major tomato exudate components (25) and strong chemoattractants of *P. fluorescens* strains (6, 26, 28); therefore, we focused the present study on the identification of a chemotaxis sensory protein(s) for amino acids in *P. fluorescens* Pf0-1. We first measured chemotactic responses of *P. fluorescens* Pf0-1 toward each of twenty commonly-occurring l-amino acids by the computer-assisted capillary assay (16). *P. fluorescens* Pf0-1 exhibited chemotactic responses toward all twenty amino acids (Table 3). In particular, Cys, Gln, Gly, Ile, Lys, Met, Phe, Pro, and Ser were strong chemoattractants of *P. fluorescens* Pf0-1, while it showed weak chemotactic responses toward Glu and Trp.

### Identification of chemotaxis sensory proteins for amino acids

In *Pseudomonas aeruginosa* PAO1, PctA, PctB, and PctC have been identified as MCPs for amino acids (12, 31). There is 46–70% identity among amino acid sequences of periplasmic domains of these MCPs. To search for the PctA homologue of *P. fluorescens* Pf0-1, BLASTP analysis was performed on a protein database of *P. fluorescens* Pf0-1 by using the 250 amino acid sequence of the putative periplasmic domain of PctA (residues 28 to 277 of PctA [accession number: NP 252999.1]) as a query sequence. BLASTP search found that three proteins, Pfl01_4431 (accession number: ABA76168), Pfl01_0124 (ABA71868), and Pfl01_0354 (ABA72098), showed the highest similarity to the query sequence (65%, 58%, and 46% identity, respectively). They possessed HCD in the C-terminal regions and have been annotated as MCPs on the basis of homology. Pfl01_4431, Pfl01_0124, and Pfl01_0354 are 59, 56, and 44% identical to the periplasmic domain of PctB (residues 27 to 277), and 49, 45, and 51% identical to that of PctC (residues 27 to 280).

To investigate whether Pfl01_4431, Pfl01_0124, and Pfl01_0354 act as MCPs for amino acids, their genes were cloned into broad-host-range plasmid pUCP18 (21), the resulting recombinant plasmids were introduced into the *pctA pctB pctC* triple mutant of *P. aeruginosa* PAO1 (*P. aeruginosa* PCT2) (31), and the recombinant strains were examined for chemotactic responses to amino acids. Whereas *P. aeruginosa* PCT2 was non-chemotactic to all amino acids tested, PCT2 (pFLCP01[containing the Pfl01_0124 gene]), PCT2 (pFLCP02 [containing the Pfl01_0354 gene]), and PCT2 (pFLCP03 [containing the Pfl01_4431 gene]) showed chemotactic responses to 16, 1, and 18 amino acids, respectively (Table 4), demonstrating that these proteins act as MCPs for amino acids. PCT2 (pFLCP01) and PCT2 (pFLCP03) showed strong responses to 5 amino acids (Ala, Asn, Lys, Met, and Ser) and 14 amino acids (Ala, Arg, Cys, Gly, His, Ile, Leu, Lys, Met, Pro, Ser, Thr, Trp, and Val), respectively, while PCT2 (pFLCP02) exhibited moderate responses only to Met. No common structural features are found among side chains of amino acids to which both PCT (pFLCP01) and PCT2 (pFLCP03) responded. Based on these results, the Pfl01_4431, Pfl01_0124, and Pfl01_0354 genes were designated *ctaA*, *ctaB*, and *ctaC* (*cta*: chemotactic transducer of amino acids).

**Fig. 1:**
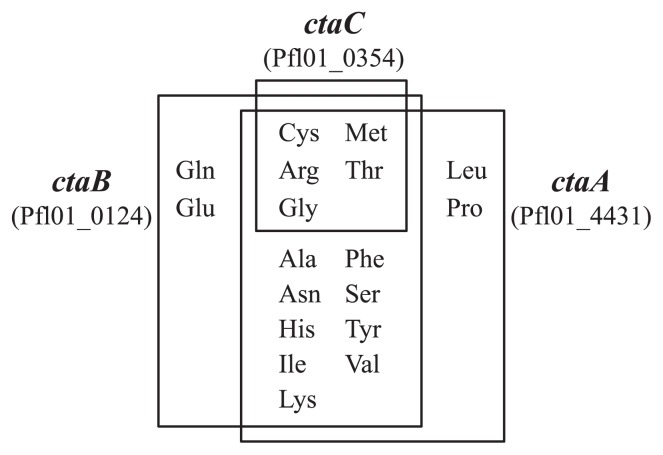
Representation of 20 commonly-occurring l-amino acids detected by chemotaxis sensory proteins. This Venn diagram is constructed based on chemotactic responses by FL0124, FL0354, and FL4431 (Table 3).

We constructed a *ctaA ctaB ctaC* triple mutant of *P. fluorescens* Pf0-1 (*P. fluorescens* FLD3) to assess the possibility of chemotaxis sensory protein(s) other than CtaA, CtaB, and CtaC. The triple mutant FLD3 showed moderate responses only toward Cys and Pro (Table 3). We then examined *ctaB ctaC*, *ctaA ctaC*, and *ctaA ctaB* double mutants of *P. fluorescens* Pf0-1 (*P. fluorescens* FL4431, FL0124, and FL0354, respectively) for their chemotaxis to amino acids to investigate the role of each MCP in amino acid chemotaxis in *P. fluorescens* Pf0-1. The *ctaA ctaB* double mutant showed a strong response to Met and Cys and weak or moderate responses to Arg, Gly, Pro, and Thr (Table 3). Since *P. fluoresces* FLD3 showed moderate responses to Cys and Pro, Met is the main chemoattractant of CtaC. The *ctaB ctaC* and *ctaA ctaC* double mutants showed strong responses to several amino acids and their response patterns were similar to those of PCT2 (pFLCP03) and PCT2 (pFLCP01), respectively. These results suggest that CtaA and CtaB play the major roles in amino acid chemotaxis in *P. fluorescens* Pf0-1 (Fig. 1).

Of the major root exudate components other than amino acids, malic acid and succinic acid were strong attractants to *P. fluorescens* Pf0-1 (data not shown); therefore, we examined *P. fluorescens* FLD3 for its ability to respond to malic acid and succinic acid. It showed chemotactic responses to malic acid and succinic acid comparable to those by the parental strain (data not shown), suggesting that CtaA, CtaB, and CtaC are not involved in the detection of malic acid and succinic acid.

### Chemotaxis of *P. fluorescens* strains to tomato root exudate

**Fig. 2:**
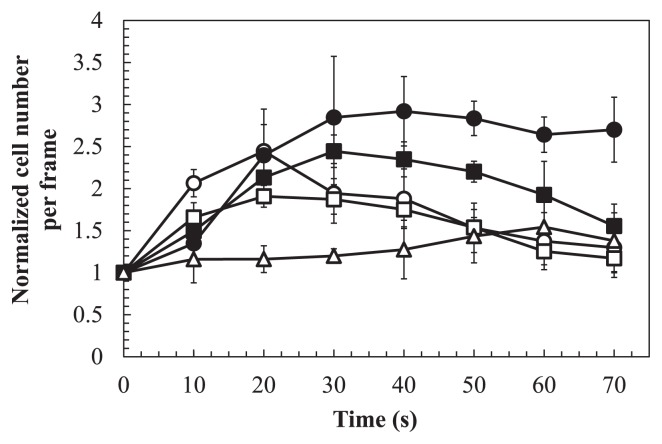
Chemotactic responses to tomato root exudate by *P. fluorescens* Pf0-1 (closed circles), FL0124 (open circles), FL0354 (open squares), FL4431 (closed squares), and FLD3 (open triangles). Digital image processing was used to count the number of bacteria around the mouth of a capillary containing exudates and 1% (w/v) agarose. One videotape frame was analyzed at each time point. The chemotactic response is presented as the normalized cell number. The normalized cell number was calculated by dividing the number of bacteria at each time point by that at the initiation of the observation. Vertical bars represent the standard deviations of measurements done in triplicate experiments.

*P. fluorescens* Pf0-1 wild-type and mutant strains were tested for chemotaxis to tomato root exudate to assess the involvement of MCPs for amino acids in chemotaxis to the root exudates. *P. fluorescens* Pf0-1 wild-type strain was strongly attracted by tomato root exudate, while *ctaA ctaB ctaC* triple mutant showed much decreased responses (Fig. 2). The double mutants showed stronger responses to root exudate than the triple mutant, but weaker responses than the *P. fluorescens* Pf0-1 wild-type strain. In particular, *ctaA ctaB* double mutant showed the weakest responses among the double mutants. This result suggests that amino acids are the major chemoattractants of *P. fluorescens* Pf0-1 in tomato root exudate. It also suggests that CtaA, CtaB, and CtaC are responsible for chemotaxis to root exudate to various degrees and that CtaC is less responsible than CtaA and CtaB.

### Root colonization analysis

**Fig. 3:**
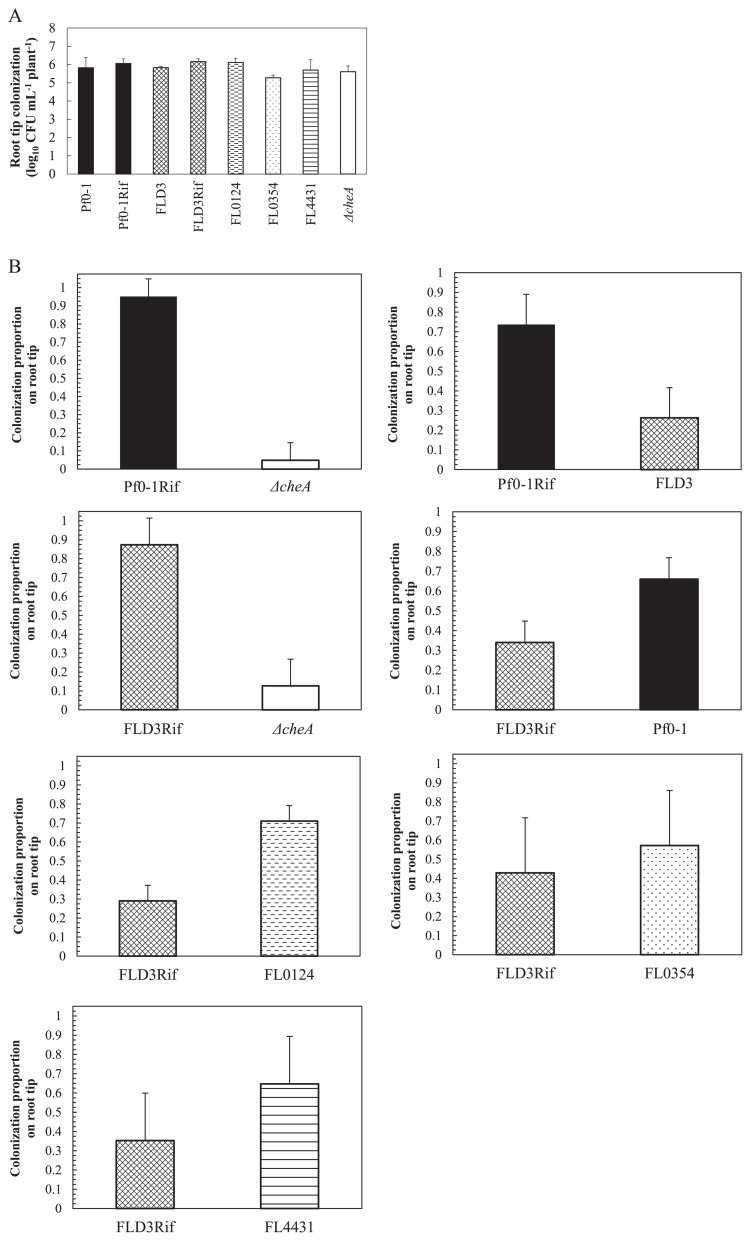
Tomato root tip colonization by *P. fluorescens* strains, (A) alone and (B) in competition with Rif ^r^ mutants. After 7 days, root systems were sampled. Bar indicates standard deviation. The total CFU numbers at the root tip in Fig. 3B were the same level as in Fig. 3A. There were significant (*P*<0.05) differences in colonization between Pf0-1Rif and Δ*cheA*, Pf0-1Rif and FLD3, FLD3Rif and Δ*cheA*, FLD3Rif and Pf0-1, and FLD3Rif and FL0124.

In order to investigate the importance of chemotaxis to amino acids in the root colonization process, we examined *P. fluorescens* Pf0-1, spontaneous rifampicin resistant mutant of Pf0-1 (Pf0-1Rif), the *ctaA ctaB ctaC* triple mutant, and its spontaneous rifampicin-resistant mutant (FLD3Rif) as well as double mutants for their root-colonizing ability by the gnotobiotic root colonization system. We also tested the *cheA* mutant of Pf0-1, which is a general non-chemotactic mutant, with the root colonization assay to confirm the report by de Weert *et al.* that flagella-driven chemotaxis is an important trait for tomato root colonization by *P. fluorescens* (6). We confirmed that there were no significant differences in growth in LB medium between mutants and the wild type Pf0-1. When germinated tomato seedlings were inoculated with single strains, all mutants colonized the tomato root to the same extent as the wild-type strain (Fig. 3A). We then carried out competitiveness assays between chemotaxis mutants and the wild-type strain by inoculating seedlings with a 1:1 mixture. Because Pf0-1Rif and FLD3Rif competed fully with Pf0-1 and FLD3, respectively (data not shown), we used Pf0- 1Rif and FLD3Rif as competitor strains in competitive colonization assays to distinguish the competing strains from tested strains. As previously shown by de Weert *et al.* (6), the non-chemotactic *cheA* mutant was a very poor competitor and showed more than 10-fold reduced ability to colonize tomato roots (Fig. 3B). The *ctaA ctaB ctaC* triple mutant exhibited higher competitive colonization ability than the *cheA* mutant, but it still showed an approximately 2-fold impaired colonization ability in the competitive colonization assay. This result indicates that chemotaxis to amino acids plays a role in the root colonization process by *P. fluorescens*. FL0124 (*ctaA ctaC* double mutant) and FL4431 (*ctaB ctaC* double mutant) showed almost 2-fold superior colonization ability than FLD3Rif, while FL0354 (*ctaA ctaB*) showed only 1.3-fold superior colonization ability than FLD3Rif. We then examined competitiveness between Δ*cheA* (*cheA* mutant) and FLD3Rif (*ctaA ctaB ctaC* triple mutant). As shown in Fig. 3B, FLD3Rif is more competitive than *cheA* mutant.

## Discussion

There are two classes of MCPs for amino acids in bacteria. One class includes Tar and Tsr of *E. coli* and *Salmonella enterica* serovar Thyphimurium, and the other includes *P. aeruginosa* PAO1 PctA. Tsr is an MCP for the attractants Ser, Ala and Gly, while Tar is an MCP for attractants Asp and Glu (29). These MCPs possess short periplasmic domains (*ca.* 150 amino acid residues), and their ligand specificity is relatively narrow. *P. aeruginosa* PAO1 PctA, which detects 18 commonly-occurring l-amino acids, shows broader ligand specificity, and its periplasmic domain (*ca.* 240 amino acid residues) is longer than those of Tsr and Tar (12). CtaA and CtaB, the main MCPs for amino acids in *P. fluorescens* Pf0- 1, detect 16 amino acids (Fig. 1, Table 3), and possess long periplasmic domains, suggesting that CtaA and CtaB belong to the class of PctA-type MCPs. BLASTP search of genome databases revealed that like *P. aeruginosa* PAO1 and *P. fluorescens* Pf0-1, other *Pseudomonas* bacteria possess 2–4 PctA-type MCPs, but not Tar/Tsr homologues. Thus, amino acids are supposed to be major chemoattractants of *Pseudomonas* bacteria.

de Weert *et al.* reported that the *cheA* mutant of *P. fluorescens* WCS365 was much less competitive for tomato root colonization than the wild-type strain and concluded that flagella-driven chemotaxis toward root exudate is an important trait for competitive root colonization (6); however, it has not been identified which chemotaxis ligands in root exudate are involved in effective root colonization. In this study, we demonstrated that *ctaA ctaB ctaC* mutant impaired in chemotaxis to amino acids showed a significant reduced ability to colonize tomato root in competitive root colonization assays using the wild-type strain as the competitor strain (Fig. 3B). This triple mutant was impaired in chemotaxis to amino acids (Table 3), but showed parental responses to malic acid and succinic acid, another major components of root exudate. These results suggest that amino acids play a role as chemoattractants for effective root colonization. Data in Table 3 and Fig. 1 suggest that CtaA and CtaB are the major MCPs for amino acids and root exudate and that CtaC contributes less to chemotaxis to amino acids and root exudate. Consistent with these data, CtaA and CtaB also make a greater contribution to the root colonization process than CtaC (Fig. 3B). In this study, we demonstrate that chemotaxis to amino acids is involved in effective root colonization by *P. fluorescens*, but it remains to be elucidated which amino acids are involved in this process.

Additionally, *ctaABC* mutant was more competitive for root colonization than *cheA* mutant (Fig. 3B), suggesting that chemoattractants other than amino acids are involved in root colonization. Since *P. fluorescens* Pf0-1 exhibits marked responses to organic acids (especially succinic acid and malic acid) (data not shown), we suppose that chemotaxis to organic acids such as succinic acid and malic acid is also involved in root colonization by *P. fluorescens*. We are now searching MCPs for organic acids in *P. fluorescens* Pf0-1 to investigate the involvement of chemotaxis to organic acids in root colonization.

## Figures and Tables

**Table 1:** Bacterial strains and plasmids used in this study

Strain or plasmid	Relevant characteristics		Reference or source
*Pseudomonas fluorescens*			
Pf0-1	wild-type strain		5
FL0124	Pf0-1 derivative, Δ*ctaA* (Pfl01_4431)	Δ*ctaC* (Pfl01_0354)	This study
FL0354	Pf0-1 derivative, Δ*ctaA* (Pfl01_4431)	Δ*ctaB* (Pfl01_0124)	This study
FL4431	Pf0-1 derivative, Δ*ctaB* (Pfl01_0124)	Δ*ctaC* (Pfl01_0354)	This study
FLD3	Pf0-1 derivative, Δ*ctaA* Δ*ctaB*	Δ*ctaC*	This study
Pf0-1Δ*cheA*	Pf0-1 derivative, Δ*cheA* (Pfl01_2038)		This study
Pf01Rif	Pf0-1 derivative, spontaneous rifampicin-resistant mutant		This study
FLD3Rif	FLD3 derivative, spontaneous rifampicin-resistant mutant, Δ*ctaA* Δ*ctaB* Δ*ctaC*		This study
*Pseudomonas aeruginosa*			
PAO1	wild-type strain		9
PCT2	PAO1 derivative, Δ*pctA* Δ*pctB* Δ*pctC*		31
*Escherichia coli*			
JM109	*recA1*, *endA1*, *gyrA96*, *thi-1*, *hsdR17* (r_k_^−^ m_k_^+^), *e14*^−^ (*mcrA*^−^), *supE44*, *relA1*, Δ(*lac-proAB*)/F′ [*traD36*, *proAB*^+^, *lacI^q^*, *lacZ* ΔM15]		18
S17-1	MM294 derivative, RP4-2 Tc::Mu-Km::Tn*7* chromosomally integrated		23
Plasmids			
pUCP18	*Escherichia-Pseudomonas* shuttle vector; Cb^r^		21
pFLCP01	pUCP18 with a 2.2 kb PCR fragment containing *ctaB* (Pfl01_0124); Cb^r^		This study
pFLCP02	pUCP18 with a 2.1 kb PCR fragment containing *ctaC* (Pfl01_0354); Cb^r^		This study
pFLCP03	pUCP18 with a 2.2 kb PCR fragment containing *ctaA* (Pfl01_4431); Cb^r^		This study
pK18*mobsacB*	Km^r^ pUC18 derivative, *lacZ*α, *mob* site, *sacB*		19
pUGMF01	pK19*mobsacB* with a 1.2-kb PCR fragment upstream of *ctaB* (Pfl01_0124) and a 1.1-kb PCR fragment downstream of *ctaB* (Pfl01_0124); Km^r^		This study
pUGMF02	pK19*mobsacB* with a 1.3-kb PCR fragment upstream of *ctaC* (Pfl01_0354) and a 1.4-kb PCR fragment downstream of *ctaC* (Pfl01_0354); Km^r^		This study
pUGMF03	pK19*mobsacB* with a 1.3-kb PCR fragment upstream of *ctaA* (Pfl01_4431) and a 1.2-kb PCR fragment downstream of *ctaA* (Pfl01_4431); Km^r^		This study

Cb^r^, carbenicillin resistance; Km^r^, kanamycin resistance.

**Table 2:** Sequence of PCR primers used in this study

Primer name	Sequence (5′ to 3′)
PFL01f	ACGTAGAATTCGAATCTGCCAGAAATGCCGTCC
PFL01r	ACGTAGGATCCGATGGGCAATGTGCTGAAGCTG
PFL02f	ACGTACTGCAGCCGAGGTGTTTGGTCGTGATAC
PFL02r	ACGTAGGATCCGTGGCGGTCGAAATAACAGCAG
PFL03f	ACGTAGGATCCAGCTAAAGGTGACAGATGCGAC
PFL03r	ACGTACTGCAGGCGTTGATCTCCCTTGGTTGAC
DPFL01Uf	ACGTACTGCAGGTAATCGAGATCCACGCTGCTG
DPFL01Ur	ACGTAGGATCCTCTGACGGCTGCTCTACTTTGG
DPFL01Df	ACGTAGGATCCGCTTCAAGATCTGATCGCTTG
DPFL01Dr	ACGTAGAATTCGATCTACATCCGCAACGGCTAC
DPFL02Uf	ACGTAGTCGACTGCGCTGCTGTTATTTCGAC
DPFL02Ur	ACGTAGGATCCATGGCAACGATCCGTATCTG
DPFL02Df	ACGTAGCATGCGAACGAATACCTGAGCCAGAAC
DPFL02Dr	ACGTAGTCGACACCTTCAGGTCGATGTATCACG
DPFL03Uf	ACGTAAAGCTTTTGTCATAACGGCCCTTGAACG
DPFL03Ur	ACGTACTCGAGCAGTCGCATCTGTCACCTTTAG
DPFL03Df	ACGTACTCGAGTCCGACGAAGACGACCAACATC
DPFL03Dr	ACGTAGAATTCCGCTCGTGAGATTCAGCCATTG
DcheAUf	AGATCTGCAGTGACGCTAAAGATCACGAAGTTGC
DcheAUr	ACTAGAATTCGTTCGGACAGTTGCTCAAGAATCTC
DcheADf	CATAGAATTCGTCAGGAAGAAGTGGTCATCAAGC
DcheADr	GCATTCTAGACGATAAGGCTTAGAACATCCATCAG

**Table 3:** Chemotactic responses of *P. fluorescens* Pf0-1 wild-type and mutant strains toward amino acids

Compounds	Chemotactic responses*				

	Pf0-1	FL0124 (Δ*ctaA ctaC*)	FL0354 (Δ*ctaA ctaB*)	FL4431 (Δ*ctaB ctaC*)	FLD3 (Δ*ctaA ctaB ctaC*)
Ala	++	+	−	+	−
Arg	++	+++	+	++	−
Asn	++	++	−	+++	−
Asp	++	−	−	−	−
Cys	+++	+++	+++	+++	++
Gln	+++	++	−	−	−
Glu	+	++	−	−	−
Gly	+++	++	+	+	−
His	++	+++	−	++	−
Ile	+++	+++	−	+++	−
Leu	++	−	−	+++	−
Lys	+++	++	−	++	−
Met	+++	+++	+++	+++	−
Phe	+++	++	−	++	−
Pro	+++	++	++	+++	++
Ser	+++	++	−	+++	−
Thr	++	+++	+	+++	−
Trp	+	−	−	−	−
Tyr	++	+	−	+	−
Val	++	+++	−	+++	−

* Videotape frames were analyzed at the initiation of observation and 1 min after the initiation. Normalized cell numbers were calculated by dividing the number of bacteria at 1 min by that at the initiation of the observation. The strength of chemotaxis is presented as the normalized cell number: +++ >3; ++ ≤3–>2; + ≤2–>1.5; −, no response.

**Table 4:** Chemotactic responses of *P. aeruginosa* PCT2 containing *P. fluorescens* Pf0-1 *ctaA*, *ctaB*, or *ctaC* genes toward amino acids

Compounds	Chemotactic responses*		

	PCT2 (pFLCP01) (+*ctaB*)	PCT2 (pFLCP02) (+*ctaC*)	PCT2 (pFLCP03) (+*ctaA*)
Ala	+++	−	+++
Arg	+	−	+++
Asn	+++	−	+
Asp	++	−	++
Cys	−	−	+++
Gln	++	−	−
Glu	+	−	−
Gly	+	−	+++
His	−	−	+++
Ile	−	−	+++
Leu	++	−	+++
Lys	+++	−	+++
Met	+++	++	+++
Phe	+	−	++
Pro	+	−	+++
Ser	+++	−	+++
Thr	+	−	+++
Trp	−	−	+++
Tyr	+	−	++
Val	++	−	+++

* Chemotactic responses are the same as in Table 3.
